# Bibliometric Analysis of the Influencing Factors, Derivation, and Application of Heavy Metal Thresholds in Soil

**DOI:** 10.3390/ijerph19116561

**Published:** 2022-05-27

**Authors:** Zhaolin Du, Dasong Lin, Haifeng Li, Yang Li, Hongan Chen, Weiqiang Dou, Li Qin, Yi An

**Affiliations:** 1 Agro-Environmental Protection Institute, Ministry of Agriculture and Rural Affairs, Tianjin 300191, China; carlcarl1988@163.com (Z.D.); lindasong608@126.com (D.L.); liy0128@163.com (Y.L.); 18702229081@163.com (H.C.); 2 Beijing Municipal Key Laboratory of Agriculture Environment Monitoring, Beijing 100097, China; haifli@126.com; 3 College of Land Science and Technology, China Agricultural University, Beijing 100193, China; dwq18513262537@126.com

**Keywords:** soil, crop, heavy metal, threshold, risk assessment

## Abstract

The study of threshold levels of heavy metals in soil is essential for the assessment and management of soil environmental quality. This study reviewed the influencing factors, the derivation, and application aspects of heavy metals’ threshold values comprehensively by a combination of bibliometric analysis and scientific knowledge mapping. A total of 1106 related studies were comprehensively extracted from the Web of Science database during the period from 2001 to 2020. The results showed that the publication output has been growing strongly. An analysis on the subject, journal, country, and institution was carried out to demonstrate the development and evolution of this research branch during the two decades. According to high-frequency keywords analysis, external factors (e.g., soil physicochemical properties) and internal factors (e.g., crop genotype) can affect heavy metal threshold values in the soil–crop system. The current methods mainly include the Point model (e.g., evaluation factor method), the Probability model (e.g., species sensitivity distribution method), and the Empirical model (e.g., ecological environment effect method). A threshold study can be applicable to the risk assessment for soil heavy metal contamination in order to determinate the soil pollution degree and its spatial and temporal distribution characteristics. Moreover, challenges and prospects of the study of heavy metal threshold values are proposed, indicating that research should focus on the relationships between human health risks and the established threshold values of heavy metals in the soil, long-term field trials and bioavailability of heavy metals for the derivation of the thresholds, and the establishment of more scientific and rational soil environmental benchmarks.

## 1. Introduction

Soil contamination has attracted widespread attention globally [1,2,3]. With the continuous advancement of urbanization and industrialization, heavy metals are constantly emitted into the terrestrial environment and accumulate in the soil, posing great threats to the quality of soil, groundwater, and crops [4,5,6]. Once heavy metals ware in the food chain, the health of humans and other organisms could be affected [7,8,9,10]. Owing to the potential toxicity and difficult biodegradation of heavy metals, their content in soil has become of great concern [9,11,12].

Threshold levels of heavy metals in soil re the maximum heavy metal concentrations that the soil can possess without destroying ecosystems [13]. The derivation of heavy metal thresholds in the soil is essential for effectively protecting soil quality, guaranteeing crop yields and quality, and ensuring human health. It also provides a scientific basis and an important foundation for ecological risk assessment and the formulation of soil environmental quality standards. Hence, it plays an indispensable role in soil management. The academic community has conducted much research on heavy metal thresholds in the soil. Some studies focused on various types of heavy metals, including certain common potentially toxic elements (e.g., cadmium (Cd)) and emerging “high-tech” critical elements (e.g., lanthanides) [13]. Some studies focused on the migration and transformation of heavy metals in soil–crop systems rather than on a simple soil system. Previous research mainly focused on protection for a sustainable use of soil [14], and current research has begun to examine the quality and safety of agricultural products as well as human health risks [15,16]. Thus, research on the thresholds of heavy metals has been extensive and diverse. However, most previous research was limited to specific situations for soil risk assessment or to qualitative analyses of small amounts of literature data. A systematic review based on big data analysis to demonstrate the development of heavy metal threshold studies is still lacking.

Bibliometric analysis is a method to summarize historical achievements and development trends comprehensively, systematically, and objectively. It can also explore possible future research directions of the research field with the help of mathematics and statistics, computer analysis and information visualization technology, combined with the analysis of the literature [17,18]. Scientific knowledge mapping, which is a scientific and effective literature research tool for large amounts of literature data to study a certain knowledge domain, is becoming increasingly popular [19]. It can vividly reveal the dynamic development of a certain research field by visualizing its development, research hotspots, and frontier knowledge [20,21].

In this study, a literature review was combined with a bibliometric analysis and scientific knowledge mapping to analyze studies on heavy metal threshold levels in soil published in the last two decades. The influence factors, derivation, and application aspects of the threshold levels were summarized through high-frequency keywords analysis. Moreover, the challenges and promising research frontiers of research on heavy metal thresholds are discussed.

## 2. Materials and Methods

### 2.1. Data Collection

Publications were obtained from the online version of the database of Science Citation Index-Expanded (SCI-E) of the Web of Science (WoS) core collection. Records retrieval started with a topic search for “soil,” “heavy metal,” and “threshold” in the publication year range from 2001 to 2020. Some repeated or irrelevant records were removed after analyzing each record. Finally, a total of 1106 records which met the study requirements were obtained.

### 2.2. Methodology of Bibliometric Analysis

CiteSpace, regarded as a useful, efficient scientific visualization tool, was used to analyze the related literature and construct scientific knowledge maps [22]. It was jointly developed by Dr. Chaomei Chen and the WISE Laboratory of Dalian University of Technology and is provided to researchers free of charge. CiteSpace V Software was used in the study for visualizing and analyzing trends and patterns in the scientific literature. Its primary source of input data was ISI WoS. It could vividly provide key information which included the publication output and journal information, country and institution cooperation, subject and research hotspot evolution through analysis functions of co-citation, collaboration and co-occurrence, respectively.

## 3. Results

### 3.1. Publication Output Analysis

As shown in Supplementary material Figure S1, the annual number of studies increased steadily from 13 in 2001 to 140 in 2020, demonstrating an almost nine-fold increase over 20 years. The predominant publication type in this field was Article, accounting for 97.92% of the studies (1083 records) [23], followed by other types such as Review (22 records) and Letter (1 record) [23,24].

### 3.2. Subject Analysis

Publications in the field of research of heavy metal thresholds in soil were distributed in 55 unique subject categories. The most productive discipline in this field was Environmental Sciences and Ecology, accounting for 67.18% of the studies (743 records), followed by Agriculture, Engineering, Water Resources, Toxicology, Geology, Public and Environmental Occupational Health, Chemistry, Plant Sciences, Science Technology, Other Topics, Geochemistry Geophysics, and Biodiversity Conservation (Table S1).

**Figure 1:**
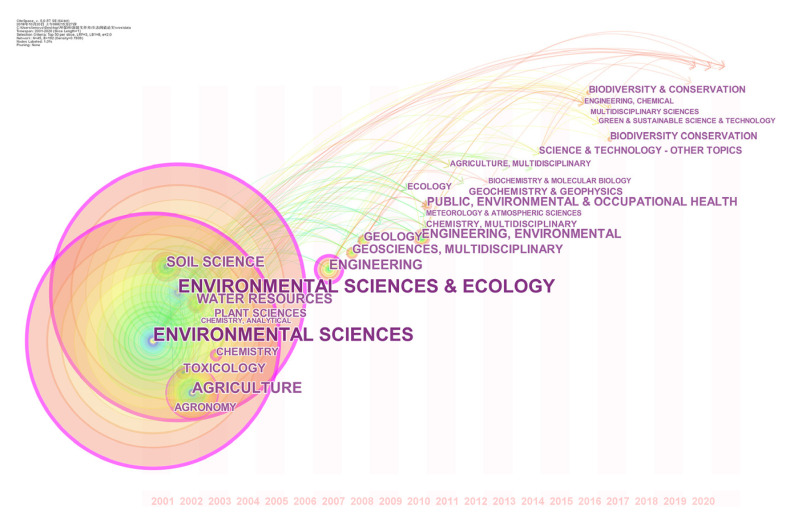
Evolution mapping of disciplines development during 2001–2020. Note: Each node represents a discipline, and the larger the node indicates the higher the number of articles related to this discipline. A pivotal point with high betweenness centrality is highlighted with a purple ring, showing this discipline has a great influence in the cooperation between disciplines.

Figure 1 shows that Environmental Science and Ecology, Environmental Science, Agriculture, Soil Science, Water Resources, Agronomy, Plant Science, Chemistry and other disciplines are basic disciplines in the research on heavy metal thresholds in soil. They have laid a solid theoretical foundation for research and development in this field. Since 2005, several disciplines including Engineering, Environmental Engineering, Geology and Geosciences, gradually became the supporting disciplines of research in this field, indicating that the research was no longer limited to theoretical research but also began to consider practical applications [25]. In 2011, the subject of “Public, Environmental and Occupational Health” appeared, which revealed that many studies began to attach importance to the relationships between the thresholds of heavy metals in soil and human health risks [26]. After 2015, certain disciplines such as “Biodiversity Conservation” and “Green and Sustainable Science and Technology” gradually became important research disciplines in this research field, demonstrating that the derivation of heavy metal thresholds in soil no longer only considered the effects on a single species but had acquired a macro view, considering the effects on whole ecosystems [27].

### 3.3. Journal, Country, and Institution Analysis

*Science of the Total Environment* was the journal with the highest number of citations, followed by *Environmental Pollution*, *Chemosphere*, and *Journal of Hazardous Materials* (Figure S2). China, the United States, and Italy were the three most productive countries. Collaborative relationships between countries were highlighted by colored lines, and a close cooperation meant that research on heavy metal thresholds in soil was still one of the most important issues and challenges worldwide (Figure S3) [22]. The Chinese Academy of Sciences, Zhejiang University, and the University of the Chinese Academy of Sciences were the top three institutions in terms of publishing volume, but Ghent University occupied a leading position in terms of the number of citations (Table S3).

### 3.4. Keyword Analysis

**Figure 2:**
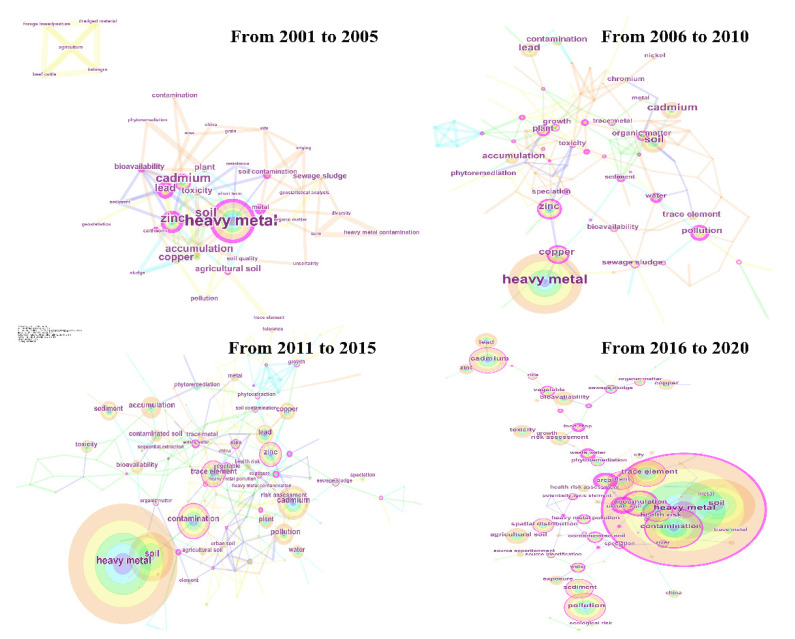
High-frequency keywords from 2001 to 2020. Note: the network is depicted with a series of tree rings in different colors, and every ring represents one keyword. The blue ring indicates the oldest keyword, and the orange ring indicates the newest. The links describe a co-occurrence of these keywords. Furthermore, pivotal points with high betweenness centrality are highlighted with a purple ring.

**Figure 3:**
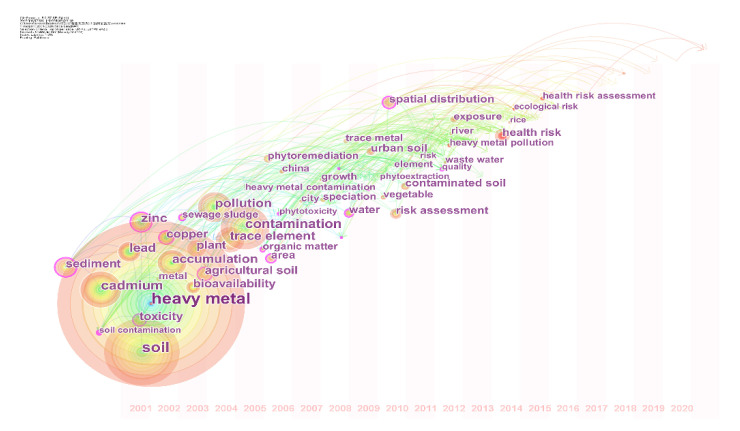
Development and evolution of high-frequency keywords. Note: each node represents a keyword, and the size of the node indicates the number of times this keyword occurred. The colored links describe the co-occurrence of keywords.

Keyword analysis was carried out in four research periods (2001–2005, 2006–2010, 2011–2015, and 2016–2020) (Figure 2). Top 50 most cited or occurring words from each period were selected as keywords, and pathfinder was used for pruning the networks. Finally, a time zone diagram of high-frequency keywords was generated to illustrate research hotspots quickly and detect the frontier direction of this research field (Figure 3). From 2001 to 2005, research paid more attention to the theoretical study of the sources and toxicity of heavy metal pollution in soil [14]. From 2006 to 2010, it was no longer limited to theoretical research but also began to attach importance to practice and applications [28]. Since 2011, ecological risk assessment became a hot research area, showing that the relationships between thresholds of heavy metals in soil and ecosystem safety started to receive great attention [15,16]. In-depth discussions in the following section were conducted based on the keyword analysis.

## 4. Discussion

### 4.1. Factors Influencing Heavy Metal Thresholds

Heavy metal pollution was often closely related to geogenic sources [29], mine waste emissions [30,31], urban and industrial wastewater [32], and agricultural practices which include excessive fertilization [33,34,35,36]. Organic or inorganic colloids played important roles in the migration of heavy metal elements in soil, and environmental physical and chemical parameters were the main influencing factors of heavy metal activation [37,38,39,40]. For example, the lower the pH of the soil, the more available heavy metals were, and the easier it was for heavy metal elements to enter plants through the soil [41,42]. The threshold values of Ni increased with the increase of the background value of Ni and clay content [43]. Different rice genotypes showed significantly different heavy metal accumulation levels in rice grains [44]. To sum up, external factors (such as heavy metal level in the soil, soil pH, redox potential, soil organic matter, and ionic strength) and internal factors (e.g., crop genotype) could affect the absorption of heavy metals in soil by crops.

### 4.2. Derivation of Heavy Metal Thresholds in Soil

From 2001 to 2005, some studies focused on the toxic effects of heavy metals in soil on different crops to explore models and methods for derivation of the thresholds [45]. For example, some research proposed that the migration of heavy metals from soil to plants was a nonlinear process, and Freundlich-type functions were recommended to define heavy metal thresholds in soil [46]. From 2006 to 2010, bioavailability models calibrated with chronic toxicity data were gradually used to formulate ecological soil standards (predicted-no-effect concentration (PNEC) values) for specific soil types, which provided an important basis for agricultural decision making [47,48]. From 2011 to 2015, research on methods and models for the derivation of threshold values for different regions and crops gradually received increasing attention [49,50]. For example, geochemical background concentration and additional risk level (maximum allowable addition amount) should be considered for the derivation of heavy metal thresholds [51]. From 2016 to 2020, the development of soil–crop transfer prediction models for the derivation of thresholds in contaminated farmland gradually became one of the research hotspots [52,53,54,55]. For example, empirical soil–plant transfer models were combined with the species sensitivity distribution (SSD) method to identify the bioaccumulation of heavy metals in crops and determine the level of heavy metal pollution in soil [56]. Overall, derivation methods of heavy metal thresholds in soil have improved with the development of multiple disciplines. The current methods include the Point model (e.g., evaluation factor method), the Probability model (e.g., SSD method), and the Empirical model (e.g., ecological environment effect method). The advantages and disadvantages of each method are shown in Table 1.

### 4.3. Application of Heavy Metal Thresholds in Soil

Based on threshold studies, risk assessment can determine a soil pollution degree and spatial and temporal distribution characteristics [64]. It could effectively allow locating areas containing toxic elements through these risk assessment procedures: setting different threshold levels for common heavy metals in soil (taking China as an example, four threshold levels of Cd were established for major edible agricultural products’ safety (e.g., rice) in consideration of soil physical and chemical properties, i.e., 0.3 mg/kg for pH ≤ 5.5, 0.4 mg/kg for 5.5 < pH ≤ 6.5, 0.6 mg/kg for 6.5 < pH ≤ 7.5, and 0.8 mg/kg for pH > 7.5), conducting more targeted investigations on heavy metal pollution characteristics, selecting appropriate methods, and defining a soil pollution classification. Hence, this analysis could play an important role in setting soil environmental benchmarks and guiding follow-up risk control measures. The common soil heavy metal pollution risk assessment methods can be divided into two categories: index and model methods [65,66]. Index methods mainly included the single pollution index method [67], the Nemerow index method [65], the pollution load index method, the environmental risk index method, and the geoaccumulation index method [68]. Model methods mainly include the enrichment factor method and the fuzzy comprehensive evaluation method. Another method is the GIS-based geostatistical legal evaluation method [69]. Index methods are relatively easy to operate, and model methods are applicable to solving complicated problems. Research mainly uses certain indicators such as enrichment factor, geoaccumulation index, Nemerow index, and average enrichment ratio to characterize the pollution degree of an area [53,70]. The content of heavy metals in crops grown on contaminated soil could also be experimentally and quantitatively evaluated with the help of the bioaccumulation factor to identify heavily polluted areas [52,55]. However, these methods could hardly determine quantitative sources of potentially toxic elements in soil management.

### 4.4. Challenges and Prospects

**Figure 4:**
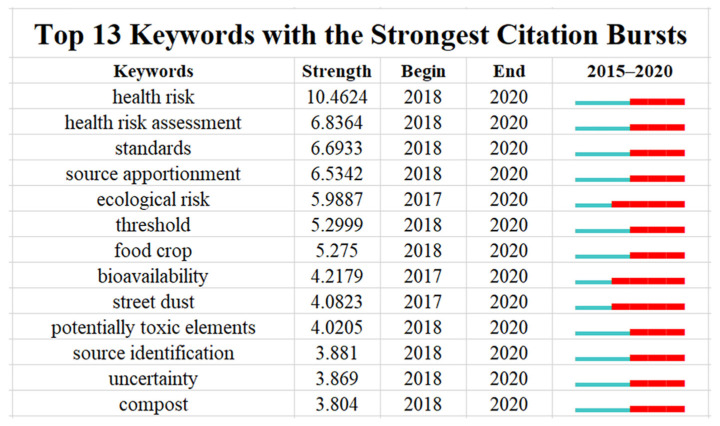
Top 13 keywords with the strongest citation bursts. Note: the duration of the citation burst of a keyword is represented by the red bar.

Frequency bursts can indicate that the scientific community has paid or is paying special attention to the contribution of specific factors, and burst detection can identify burst keywords as indicators of emerging trends [71]. As shown in Figure 4, the discussion of the factors influencing heavy metal thresholds in soil will continue in the future, especially concerning the three aspects of “health risk assessment”, “ecological risk assessment”, and “apportionment and identification of pollution sources” [72]. The topics of “health risk” and “health risk assessment” have emerged as research frontiers, with the strongest strength of frequency bursts corresponding to 10.4624 and 6.8364, respectively, indicating that this research will be of interest to scientists in the future. Performing a comprehensive ecological risk assessment plays a remarkable role in formulating scientific, reasonable heavy metal thresholds in soil, but specific adaptation and accuracy of ecological risk assessment remain a challenge. Methods and tools for identifying and analyzing sources of heavy metal pollution still need further research. The current highly complex and changeable environmental situation and the considerable effects on soil pollution of human activities make heavy metal pollution sources difficult to identify. this may also be one of the reasons why the terms “source apportionment” and “source identification” have become burst keywords.

#### 4.4.1. Relationships between Human Health Risks and Heavy Metal Thresholds in Soil

Heavy metals in soil can enter the food chain after enrichment in crops, which can pose a severe threat to human health. Moreover, human beings accumulate heavy metals also through inhalation of particles in soil and by dermal contact [15]. Under the combined effects of various exposure pathways, the derivation of heavy metal thresholds in soil requires multidisciplinary joint research. Therefore, it is worthy to explore how to establish heavy metal thresholds in soil, ensuring that these contaminants do not cause risks to human health in the future.

#### 4.4.2. Derivation of Heavy Metal Thresholds Based on Long-Term Field Trials

The current studies mostly use short-term pot experiments or field trials to understand quantitative relationships regarding heavy metal concentrations in soil–crop systems. Large spatiotemporal differences in the experimental conditions between pot experiments and field trials often lead to inconsistent experimental results. Consequently, heavy metal thresholds in soil often failed to meet the requirements of soil pollution risk assessment, with relatively poor reliability. Determining quantitative relationships and studying the dynamic changes of heavy metal thresholds in long-term field trials may become future research topics.

#### 4.4.3. Derivation of Heavy Metal Thresholds Based on Their Bioavailability in Soil

At present, methods for determining heavy metal thresholds in farmland soil mainly consider the total amount of heavy metals as evaluation standards. However, the total amount of heavy metals cannot fully represent the actual toxic effects on crops and humans. The pH, organic matter content, and other factors should be considered for the derivation of heavy metal thresholds, and this requires research innovations. Therefore, the construction of new derivation methods for thresholds based on the bioavailable state of heavy metals in the soil may be an important development direction.

#### 4.4.4. Establishment of Soil Environmental Benchmarks 

Soil environmental benchmarks based on heavy metal thresholds can scientifically reflect the risk of heavy metal contamination in soil on related receptors. They can guide the formulation of soil environmental quality standards and subsequent pollution risk assessment. However, the current soil environmental benchmarks in many countries are hard to meet; therefore, there is a need to scientifically formulate soil environmental quality standards. For example, heavy metal threshold values based on different pH levels and land-use types were established in the China’s current farmland soil environmental quality standard (GB15618-2018). This standard can be beneficial to determine different farmland soil pollution levels preliminarily. However, this standard did not take into account other factors influencing heavy metal thresholds, such as soil types, soil physical and chemical properties (such as redox potential, soil organic matter, and ionic strength), and crop varieties. As a result, it may cause risk assessment results to deviate from reality, which can lead to “overprotection” or to “inadequate protection” in relation to soil environmental quality safety. Therefore, establishing more scientific and rational soil environmental benchmarks regarding heavy metal thresholds in soil may be important research direction.

## 5. Conclusions

Based on a big data analysis of 1106 studies, the following results on heavy metal thresholds in soils were obtained: (1) Environmental Sciences and Ecology, Agriculture, and Engineering were the three main disciplines concerned with heavy metal threshold research. (2) Journals, countries and institutions with the highest number of articles published were identified, and the development and evolution of these studies during the two decades were demonstrated. (3) Systematical analysis of external influencing factors (such as heavy metal levels in soil, soil pH, redox potential, soil organic matter, and ionic strength) and internal factors (e.g., crop genotype), of derivation methods (such as point model, probability model, and empirical model), and of application aspects of heavy metal threshold studies from 2001 to 2020 was carried out. (4) Challenges and future prospects of heavy metal threshold studies were proposed, indicating that research should focus on the relationships between human health risks and the established thresholds of heavy metals in soil, the derivation of thresholds based on the bioavailability of heavy metals and long-term field trials, and the establishment of more scientific and rational soil environmental benchmarks.

## Figures and Tables

**Table 1:** Analysis on the advantages, disadvantages, and uncertainty of the methods for determining the threshold levels of heavy metals in agricultural land.

Model	Representative Method	Advantages and Disadvantages	References
Point model	Evaluation factor method	Advantages: simple and easy to operate, taking account of species sensitivity, independent of any theoretical model; quantitative result description Disadvantages: selection of evaluation factors depending on experiences and national policies, single type and quantity of sensitive species without representativity	[57,58]
Probability model	Species sensitivity distribution (SSD)	Advantages: taking account of differences in species sensitivity, soil physical and chemical properties, bioavailability and sources of pollutants, and different pollution risk levels; quantitative result description Disadvantages: rarely taking account of the interrelationship of food chains between species, unable to provide potential recovery information for the environment	[58,59,60]
Empirical model	Ecological environment effect method	Advantages: taking account of the effects of different soil physical and chemical properties; quantitative result description Disadvantages: rarely taking account of species sensitivity	[59,61,62,63]

## Data Availability

Not applicable.

## Supplementary Materials

The following supporting information can be downloaded at: https://www.mdpi.com/article/10.3390/ijerph19116561/s1, Figure S1: Annual number of various types of publications (2001–2020). Note: the bar chart shows the annual number of various types of publications, and the line chart shows cumulative frequency; Figure S2: Most cited journals. Note: each node represents a journal, and a brighter node indicates this journal is highly cited; Figure S3: Development and evolution of the studies in the most productive countries. Note: each node represents a country, and a larger node indicates a higher number of articles published by this country. The pivotal point with high betweenness centrality is highlighted with a purple ring, showing that country has a great influence in the cooperation relationships between countries; Table S1: Twelve most productive disciplines; Table S2: Six most productive journals; Table S3: Fifteen most productive institutions.
