# Supplementary material for: Bibliometric Analysis of the Influencing Factors, Derivation, and Application of Heavy Metal Thresholds in Soil
Source: Int J Environ Res Public Health. 2022 May 27;19(11):6561. doi: 10.3390/ijerph19116561 (PMC9180750; doi:10.3390/ijerph19116561)
Supplement: Supplementary file 1 [file ijerph-19-06561-s001.zip › Highlights.pdf]

Highlights:

- Advances of heavy metal threshold of soil were analyzed by bibliometrics.
- Methods to establish heavy metal threshold of soil were summarized.
- Current challenges and future prospects of threshold study were discussed.
