# Supplementary material for: Bibliometric Analysis of the Influencing Factors, Derivation, and Application of Heavy Metal Thresholds in Soil
Source: Int J Environ Res Public Health. 2022 May 27;19(11):6561. doi: 10.3390/ijerph19116561 (PMC9180750; doi:10.3390/ijerph19116561)
Supplement: Supplementary file 1 [file ijerph-19-06561-s001.zip › Supplementary material-clean version.pdf]

Bibliometric Analysis on the Influence Factors, Derivation and application of Heavy Metal Threshold of Soil

Zhaolin Du [1], Dasong Lin [1], Haifeng Li [2], Yang Li [1], Hongan Chen [1], Weiqiang Dou [3], Li Qin [1,*], Yi An [1,*]

[1] Agro-Environmental Protection Institute, Ministry of Agriculture and Rural Affairs, Tianjin 300191, China; carlcarl1988@163.com (Z.D.); lindasong608@126.com (D.L.); LiY0128@163.com (Y.L.); 18702229081@163.com (H.C.)

[2] Beijing Municipal Key Laboratory of Agriculture Environment Monitoring, Beijing, 100097, China; haifli@126.com (H.L.)

[3] College of Land Science and Technology, China Agricultural University, Beijing 100193, China; dwq18513262537@126.com (W.D.)

* Correspondence: ql-tj@163.com (L.Q.); simon8601@126.com (Y.A.)

1. Publication output analysis

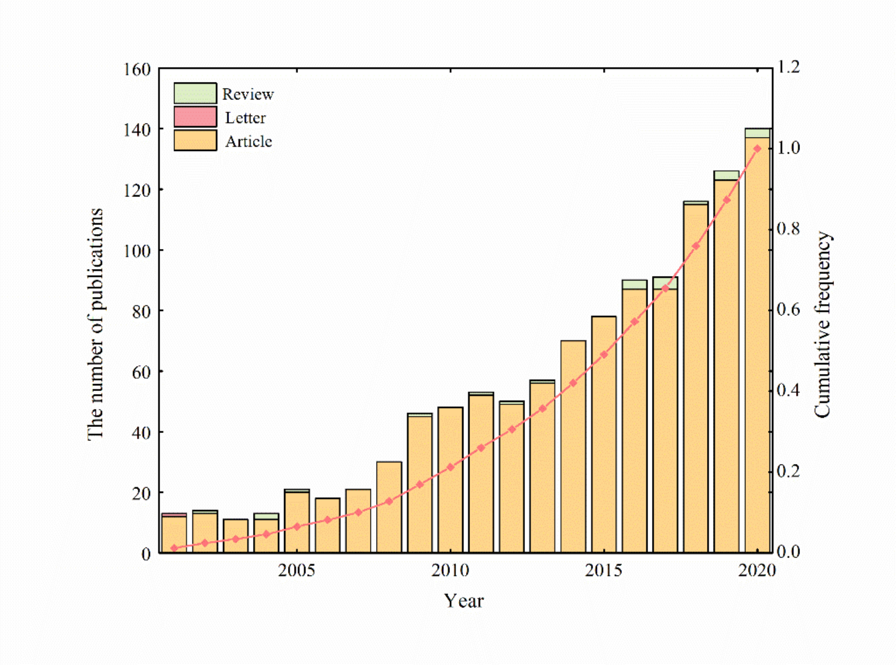

Figure S1. Annual number of various types of publications (2001-2020). Note: The bar chart shows the annual number of various types of publications, and the line chart shows cumulative frequency.

2. Subject analysis

Table S1 Twelve most productive disciplines.

Subject Categories	R	TP	%
Environmental Sciences & Ecology	1	743	67.18%
Agriculture	2	182	16.46%
Engineering	3	106	9.58%
Water Resources	4	102	9.22%
Toxicology	5	74	6.69%
Geology	6	69	6.24%
Public & Environmental Occupational Health	7	55	4.97%
Chemistry	8	49	4.43%
Plant Sciences	9	47	4.25%
Science Technology-Other Topics	10	31	2.80%
Geochemistry Geophysics	11	30	2.71%
Biodiversity Conservation	12	29	2.62%

Note: R: the ranking of the total number of publications; TP: the total number of publications; %: the percentage of the total number of publications.

3. Journal analysis

These 1106 publications were published in a wide range of 297 journals, among which the six most productive journals (TP>30) published 285 articles (25.8%) (Table S2). Science of the Total Environment published the most articles (70, 6.3%) with the large academic influence (IF of 6.551 in 2019). Other productive journals include Environmental Science and Pollution Research, Environmental Monitoring and Assessment, Environmental Pollution, Chemosphere, and Ecotoxicology and Environmental Safety, and provide a useful reference for scholars in this field to read related literature or publish related articles.

The most highly cited journals in this field were also analyzed (Figure S2). Each node in the figure represents a journal, and a brighter node indicates that this journal is highly cited. The top-ranked journal by citation count is Science of the Total Environment, with 2704 records, followed by Environmental Pollution (2070 records), Chemosphere (1640 records), and Journal of Hazardous Materials (1018 records). These journals with a higher citation count are often the main sources of cited works for the research in this field with most influential contributions to the development of the field.

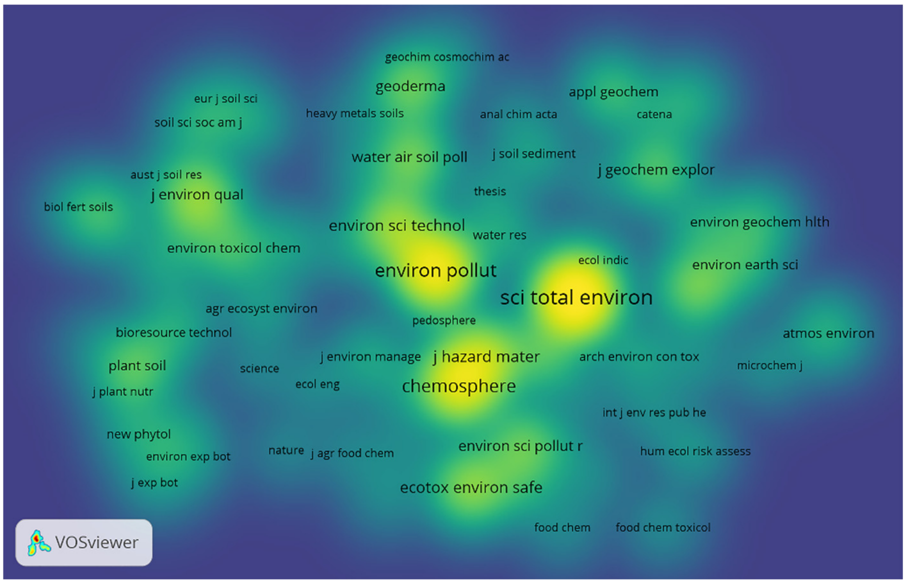

Figure S2. Most highly cited journals. Note: Each node represents a journal, and a brighter node indicates this journal has highly cited.

Table S2 Six most productive journals.

Journal Name	R	TP	%	IF 2019	h-index
Science of the Total Environment	1	70	6.3	6.551	205
Environmental Science and Pollution Research	2	62	5.6	3.056	82
Environmental Monitoring and Assessment	3	44	4.0	1.903	91
Environmental Pollution	4	42	3.8	6.793	194
Chemosphere	5	35	3.2	5.778	212
Ecotoxicology and Environmental Safety	6	32	2.9	4.872	110

Note: R: the ranking of the total number of publications; TP: total number of publications; %: the percentage of the total number of publications; IF: impact factor.

4. Country analysis

The development and evolution of countries that have made more substantial contributions to the research on the thresholds of heavy metals in soil are shown in Figure S3. The size of the node is proportional to the number of publications; hence, the number of publications written by the Chinese is far ahead, reaching 302 and accounting for 27.31% of the total publications, followed by the United States (97, 8.77%), Italy (83, 7.50%), and Spain (72, 6.51%). The dark shade of the inner circle of the node indicates that this country's research started earlier. The United Kingdom started to study this research field earlier, followed by the United States, Germany, Spain, and China. Additionally, the purple outer ring of the node reflects the influence of the country's cooperative relations. The figure shows that most of the nodes with larger purple outer rings are European countries, the United States, and China, indicating that these countries may play a more important role in the cooperation with other countries.

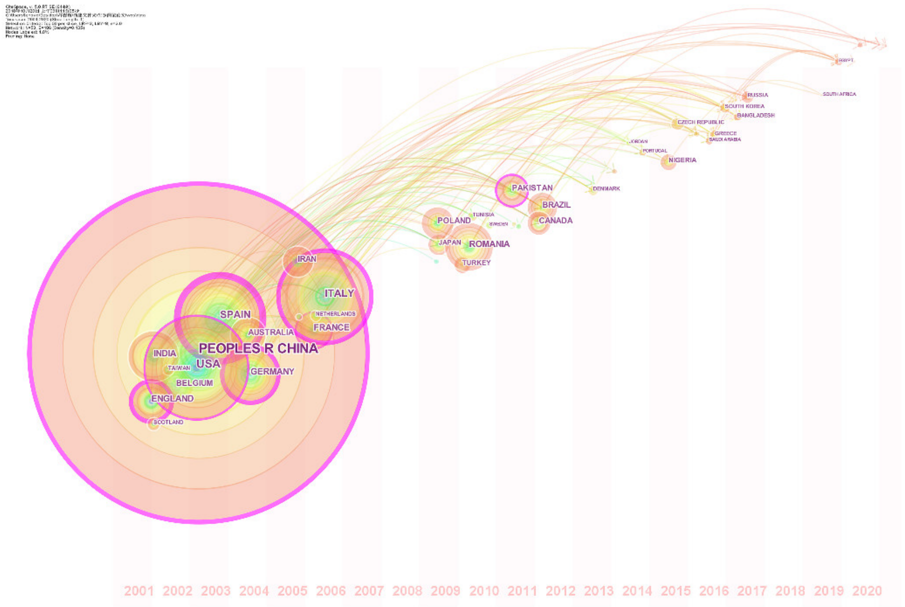

Figure S3. Development and evolution of the most productive countries. Note: Each node represents a country, and a larger node indicates this country published more articles. Pivotal point with high betweenness centrality is highlighted with a purple ring, showing this country has a great influence in the cooperation relationships between countries.

5. Institution analysis

Table S3 Fifteen most productive institutions.

Institutions	Number of publications	Total citations of publications	Average citations of per publication
Chinese Academy of Sciences	66	1227	18.59
Zhejiang University	23	1095	47.61
University of Chinese Academy of Sciences	21	232	11.05
Beijing Normal University	19	501	26.37
Chinese Academy of Agricultural Sciences	19	245	12.89
Ghent University	17	933	54.88
Katholieke Universiteit Leuven	15	566	37.73
Chinese Research Academy of Environmental Sciences	13	195	15.00
Consejo Superior de Investigaciones Científicas	12	376	31.33
University of Florida	11	376	34.18
University of Naples Federico II	11	157	14.27
University of Bologna	11	136	12.36
National Taiwan University	10	355	35.50
University of Barcelona	10	260	26.00
Lanzhou University	10	175	17.50

Table S3 lists the basic information of the research institutions with more than 10 articles. The top 15 institutions in this field were from China, Belgium, Spain, Italy, and the United States, of which Chinese research institutions account for more than half. Sorted by the total number of publications, the Chinese Academy of Sciences has the most publications, reaching 66, followed by Zhejiang University (23) and the University of Chinese Academy of Sciences (21). In terms of average citations per article, the Ghent University occupies a leading position, showing that its research results may be of great importance to the development of this field. Although the Chinese Academy of Sciences ranks first in the number of articles published, the number of citations is not very high. Therefore, it still needs to improve the quality of its articles further to strengthen its international academic influence.
